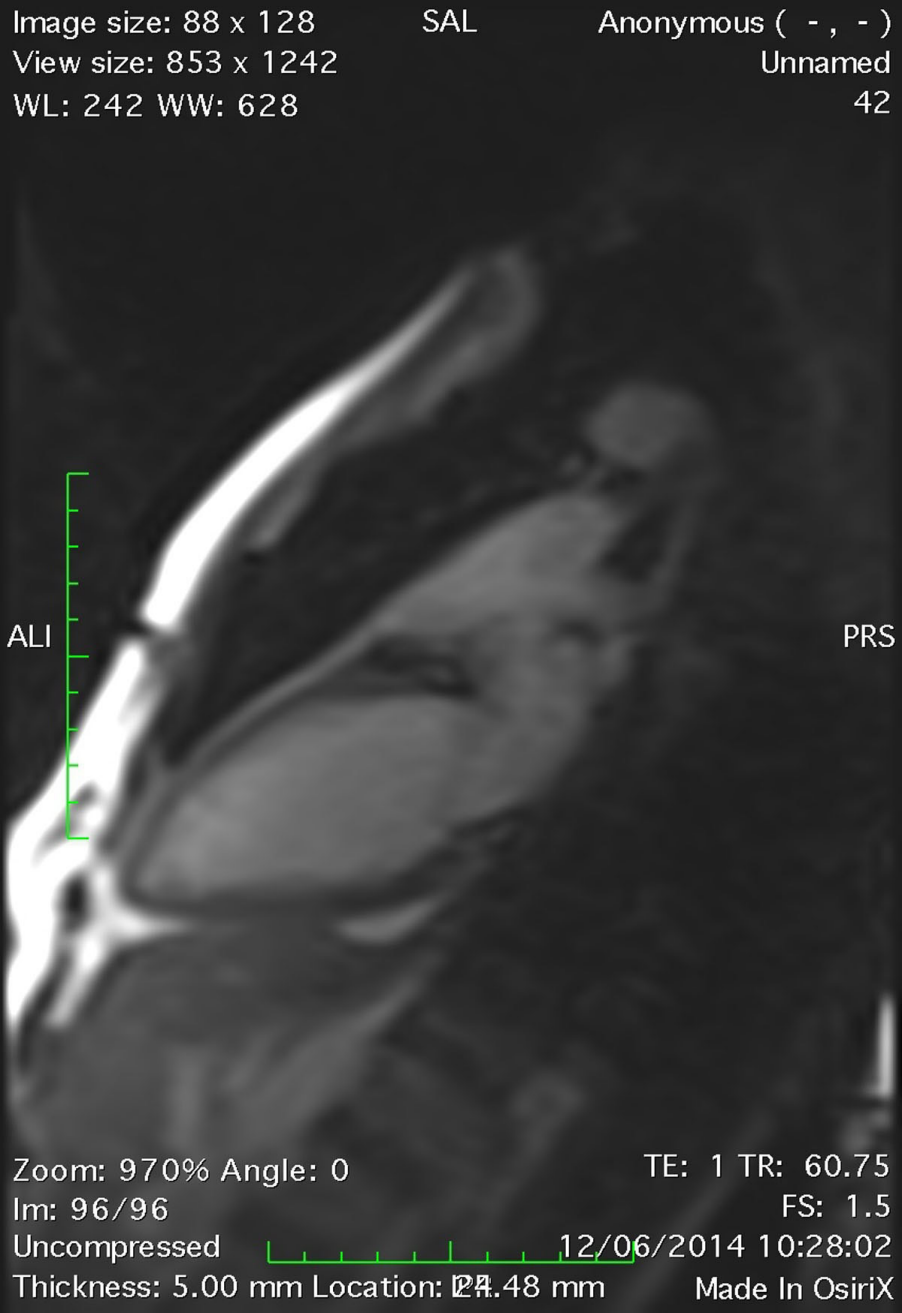# Measuring ventricular volumes during exercise with vertical long axis cines

**DOI:** 10.1186/1532-429X-17-S1-P386

**Published:** 2015-02-03

**Authors:** Gergely V Szantho, Stephen Lyen, Chris B Lawton, Nathan E Manghat, Chiara Bucciarelli-Ducci, Mark Hamilton

**Affiliations:** 1 Bristol Heart Institute, Bristol, UK; 2 University of Bristol, Bristol, UK

## Background

Imaging cardiac function during exercise remains difficult. Some researchers interrupted the exercise to obtain data, but this is not physiological. Others used real time short axis cine imaging, which presents issues with respiratory motion. La Gerche et al (Circ. Cardiovasc. Imaging 2013) may have solved this by linking the short axis images to the respiratory cycle with additional long axis imaging, but using a not widely available in-house software.

We set out to explore a feasible alternative method of scanning and analysis.

## Methods

A contiguous stack of real time cine steady state free precession (slice thickness 5mm, no gap, temporal resolution 60ms, voxel size ~2.8x4.6x5mm) images through the whole heart was performed in the vertical long axis (VLA) plane. VLA is perpendicular to the diaphragm, so the diaphragmatic motion and the breathing cycle can be followed. The VLA plane shows the atrioventricular border, hence reduces error. Resting VLA volumes were compared to standard, gated, short axis volumes.

Eight healthy volunteers underwent continuous aerobic leg exercise with MR-compatible ergometer (Up/Down, Lode, Groningen, Netherlands) in the MRI scanner (Siemens Avanto 1.5T). Imaging was carried out at rest (10 second/slice to include a full breathing cycle in each cine) and during two stages of exercise, at target heart rates of 150% and 180% of resting. The resistance of the ergometer was gradually increased to meet target heart rate, maintaing at least 60 rpm. Inspiratory and expiratory cardiac cycles could manually be selected with following the diaphragm.

## Results

All images were diagnostic regarding endocardium detection, atrioventricular valve position and breathing cycle detection (*Image*)*.* Standard volumes correlated well with real-time VLA volumes (LVEDV 169.73+-39.51ml vs 210.39+-40.36ml r=0.975 p< 0.0005, LVESV 68.13+-21.91ml vs 84.97+-16ml r=0.956 p<0.001, LVSV 101.6+-18.91ml vs 125.42+-26.97ml r=0.931, p<0.005). Physiological response was observed:

1) Ejection fraction of both ventricles increased significantly from rest through moderate to higher level of exercise, either measured on inspiration or expiration (from 7 to 13 percentage points, p<0.05 in each cases).

2) Left ventricular stroke volume increased significantly from rest to moderate and from rest to high level of exercise on expiration, 125.42+-26.97ml vs 135.56+-23.35ml (p<0.005) and 141.71+-34.78 (p<0.02), respectively.

3) A difference between RV stroke volume on inspiration (RVSVinsp) and RV stroke volume on expiration (RVSVexp) became significant at peak exercise (RVSVinsp 136.24+-43.09ml vs RVSVexp 115.98ml+-34.62, p<0.02).

The latter two findings may show the effect of the respiratory pump on the circulation.

## Conclusions

Our pilot study shows that it is feasible measuring ventricular volumes during exercise, using a widely available sequence and without the need for additional customised software. Further work is being undertaken to confirm these results in a large scale population.

## Funding

Funded by the general research budget of our Institution.

**Figure 1:**